# Supplementary material for: Correct determination of charge transfer state energy from luminescence spectra in organic solar cells
Source: Nat Commun. 2018 Sep 7;9:3631. doi: 10.1038/s41467-018-05987-8 (PMC6128889; doi:10.1038/s41467-018-05987-8)
Supplement: Supplementary file 1 — Supplementary Information [file 41467_2018_5987_MOESM1_ESM.pdf]

Supplementary Information

Correct Determination of Charge Transfer State Energy from Luminescence Spectra in Organic Solar Cells

Mathias List[1], Tanmoy Sarkar[1], Pavlo Perkhun[2], Jörg Ackermann[2], Chieh Luo[1] and Uli Würfel[1]

[1]Fraunhofer Institute for Solar Energy Systems (ISE), Heidenhofstr. 2, 79110 Freiburg, Germany. [2]Centre Interdisciplinaire de Nanoscience de Marseille (CINaM), Campus de Luminy, Case 913, 13288, Marseille, France. Correspondence and requests should be addressed to U.W. (email: uli.wuerfel@ise.fraunhofer.de)

Supplementary Figures

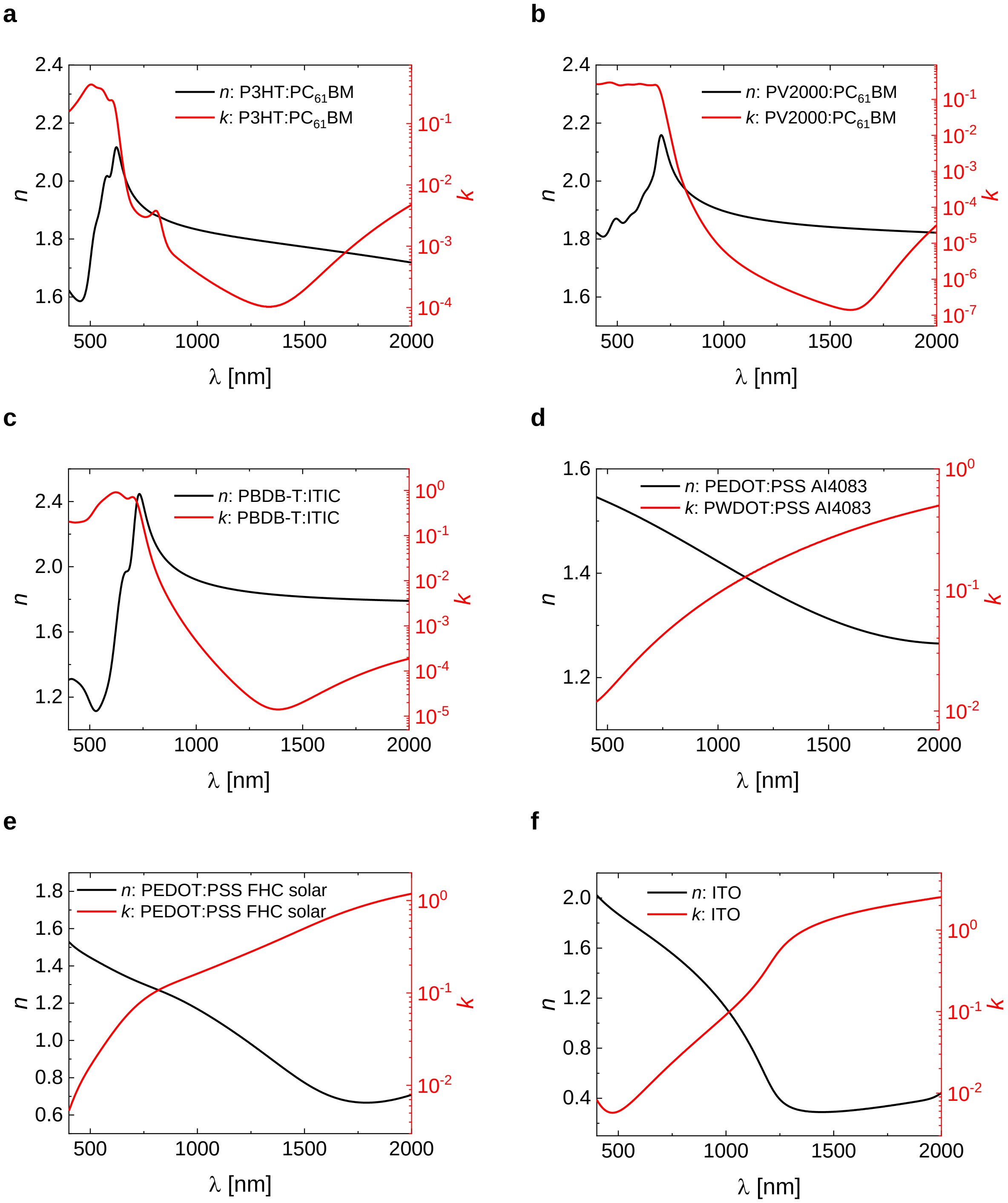

Supplementary Fig. 1: Refractive index n and extinction coefficient k of: P3HT:PC_{61}BM in (**a**), PV2000:PC_{61}BM (**b**), PBDB-T:ITIC (**c**), of PEDOT:PSS Clevios™ AI 4083 (**d**), PEDOT: PSS Clevios™ F HC Solar (**e**), and indium tin oxide (ITO) in **f**.

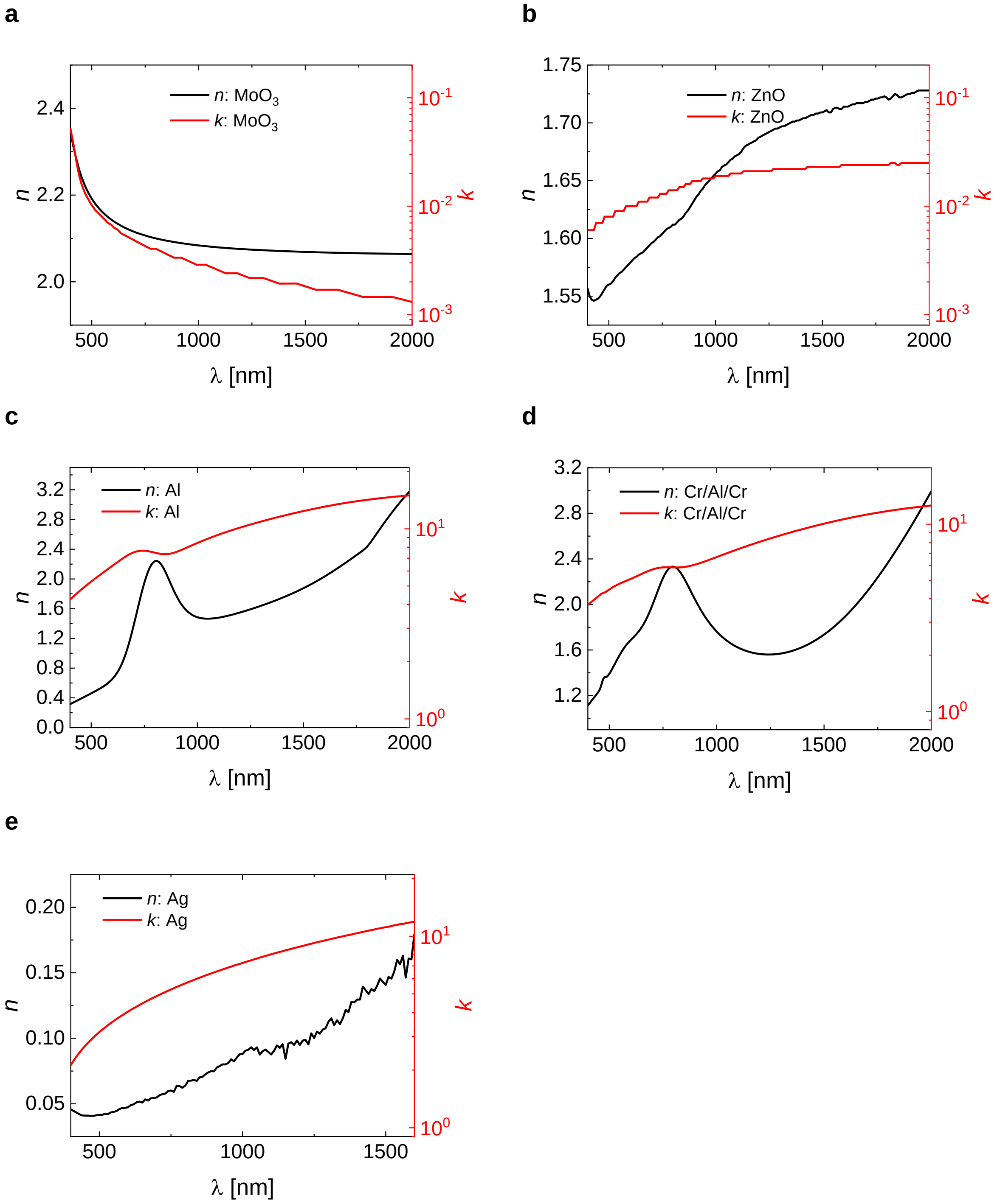

Supplementary Fig. 2: Refractive index n and extinction coefficient k of molybdenum trioxide (MoO_3) [1] (**a**), zinc oxide (ZnO) in **b** [2], aluminum (**c**), chromium/aluminum/chromium (Cr/Al/Cr) in **d** and silver (Ag) in **e** [3].

Supplementary References

[1] L. Lajaunie, F. Boucher, R. Dessapt, P. Moreau, Strong anisotropic influence of local-field effects on the dielectric response of α -MoO_3, Phys. Rev. B 88 (2013).

[2] M.R. Querry, Report No. D-A158-623: Optical Constants, University of Missouri (1985).

[3] K.M. McPeak, S.V. Jayanti, S.J.P. Kress, S. Meyer, S. Iotti, A. Rossinelli, D.J. Norris, Plasmonic Films Can Easily Be Better: Rules and Recipes, ACS Photonics 2 (2015) 326–333.
